# *TCF7* ‐Linked Immune Resilience: A Salutogenic Biological Warranty for Longevity

**DOI:** 10.1111/acel.70090

**Published:** 2025-04-28

**Authors:** Rituparna Ghosh, Matthew J. Yousefzadeh

**Affiliations:** ^1^ Burch‐Lodge Center for Human Longevity, Columbia Center for Translational Immunology Columbia University Medical Center New York New York USA

**Keywords:** aging, cellular immunology, inflammation, senescence

## Abstract

Immune resilience as a salutogenic force to preserve healthspan and resilience to challenges. The figure was created with BioRender (https://www.BioRender.com/).

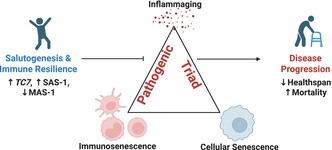

Aging is a complex but malleable phenomenon that is not fully understood. Although individuals have historically aged at similar rates, there is dramatic variability in healthspan within individuals of a similar chronological age. Numerous models of aging have been proposed to explain these differences, but most models traditionally focus on disease and dysfunction. These models largely overlook salutogenesis, which emphasizes factors that promote health and well‐being throughout the life course. Nearly 45 years ago, sociologist Aaron Antonovsky introduced salutogenesis, describing intrinsic characteristics that enhance resilience to stress and improve the capacity to adapt to aging or disease (Antonovsky 1980). This framework has the potential to be transformative for aging research, as recent findings from large human cohort studies explore immune resilience and its impact on overall health and outcomes.

The recent publication “The 15‐Year Survival Advantage: Immune Resilience as a Salutogenic Force in Healthy Aging” we gain insight into the intersection of immune function, longevity, and aging through the concept of immune resilience (IR) (Manoharan et al. 2025). Throughout evolution proinflammatory processes play a critical role in ensuring host responses to enhance survival, however the ability to control inflammation diminishes with age, increasing the risk of disease and mortality. This decline is exemplified by inflammaging, the process by which older individuals develop low‐grade chronic inflammation that can accelerate biological aging. In this recent publication, the authors show that IR integrates both immunocompetence (the ability to mount effective responses to challenges) and the capacity to regulate inflammation. IR requires the integration of both the innate and adaptive immune systems to counter the “pathogenic triad” of chronic inflammation (inflammaging), immune aging (immunosenescence), and the accumulation of senescent cells (Figure 1). The authors hypothesize that premature mortality is due to failure to maintain salutogenic adaptations in lieu of intrinsic biological constraints of aging.

**FIGURE 1:**
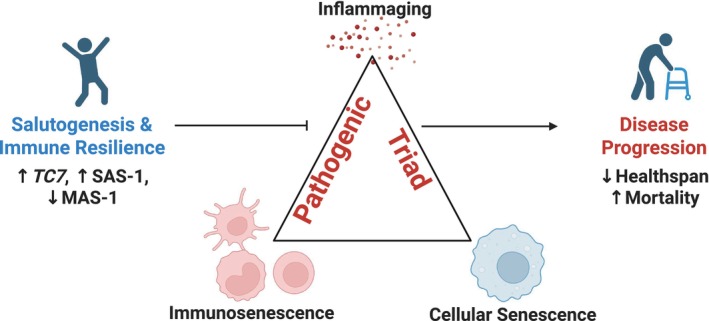
Immune resilience as a salutogenic force to preserve healthspan and resilience to challenges. The figure was created with BioRender (https://www.BioRender.com/).

Previously, the authors demonstrated that IR, the capacity to preserve or rapidly restore immune function to promote disease resistance and limit inflammation, could be assessed using transcriptomic analysis of peripheral blood cells (Ahuja et al. 2023). This analysis led to the creation of two IR signatures: the survival‐associated signature (SAS‐1) and the mortality‐associated signature (MAS‐1). Higher levels of SAS‐1 were associated with increased immunocompetence and reduced inflammation, while elevated MAS‐1 was associated with poor immunocompetence and enhanced inflammation, typical of inflammaging (Ahuja et al. 2023). This study highghlights a novel and powerful salutogenic trait: the *TCF7*‐linked IR trait (Manoharan et al. 2025). Characterized by high expression of T‐cell factor 7 (*TCF7*), whose gene product TCF1 functions via the Wnt pathway, is essential for the development and maintenance of stemness in T cells (Sturmlechner et al. 2023; Zhao et al. 2022). The authors found that high *TCF7* expression is a component of the SAS‐1, and individuals with or capable of restoring this trait showed attenuated aging features compared to those who lack this key salutogenic component. Based upon their findings, maintaining high *TCF7* expression was critical to sustaining immune resilience (SAS‐1^high^ MAS‐1^low^) (Manoharan et al. 2025). The authors demonstrate that individuals across the lifespan who maintain or restore this trait exhibit attenuated features of aging, including inflammaging, immune aging, and senescence. This discovery aligns with the principles of salutogenesis, suggesting that optimal immune function—rather than merely the absence of disease—plays a key role in promoting health and longevity.

Interestingly, markers of salutogenesis (*TCF7* expression, SAS‐1, and MAS‐1 transcriptomic signatures) showed a weak correlation with chronological age, suggesting that the mechanisms governing IR are, at least in part, age‐independent. This finding is exciting because it indicates that both age‐dependent and ‐independent mechanisms can regulate IR and the pathogenic triad. Single‐cell analyses provided further insights: the SAS‐1 profile, along with positive changes in salutogenic pathways and *TCF7* expression, were differentially expressed in dendritic cells (higher levels) and CD8^+^ T cells (lower levels), while the deleterious MAS‐1 profile was preferentially enriched in monocyte populations. Natural killer (NK) cell‐enriched signatures, however, had no demonstrable effect on lifespan, implying that NK cell activity may not be a primary determinant of lifespan in the context of IR. The single‐cell transcriptomics findings reinforce that different immune cell types play distinct roles in governing immunocompetence and inflammation control (Manoharan et al. 2025).

The study underscores the evolutionary significance of immune resilience. Inflammation, a double‐edged sword, is essential for survival but also contributes to the development of diseases and mortality. Understanding how evolutionary pressures have shaped our immune system to balance inflammation's protective and harmful effects offers profound implications for aging. The authors propose that individuals who preserve or reconstitute optimalimmune function are better equipped to navigate the challenges of age‐ related health issues.

IR, particularly through higher *TCF7* expression, experience greater survival advantages, lower comorbidities, and reduced mortality risk. Using several case studies (IR status and responses to infections, vaccinations, myocardial ischemia, etc.), the authors were able to stratify the IR trajectories of subjects into three different groups with unique biological properties: IR‐preservers (robustness), IR‐reconstituters (plasticity), and IR‐degraders (maladaptation). IR‐preservers maintained robust immunocompetence and resisted the development of the pathogenic triad, while IR‐reconstituters experienced a transient decline in optimal IR‐*TCF7* ^high^ status that was eventually restored upon convalescence. Notably, IR‐preservers and IR‐reconstituters were indistinguishable after recovery, as both groups presented with the optimal IR‐*TCF7* ^high^ status. Conversely, IR‐degraders failed to re‐establish high *TCF7* expression, eventually developing persistent presentation of the pathogenic triad. Based upon these findings individuals who preserve optimal IR should possess: (1) robust pathogen defense across a broad spectrum of challenges, (2) distinctive survival‐associated immune/inflammatory signatures, and (3) superior humoral responses. Their findings demonstrate that this IR trait confers a 69% reduction in mortality in non‐COVID‐19 individuals and provides survival advantages in COVID‐19 cohorts, with the most pronounced benefits seen in individuals under 70 years. In fact, 40‐year‐old subjects that are extreme IR degraders (with a SAS‐1^low^ MAS‐1^high^ profile) had a sex‐adjusted hazard ratio over 9 compared to age‐matched subjects with optimal IR (SAS‐1^high^ MAS‐1^low^). Their mortality risk was equivalent to that of 55‐year‐olds with an optimal IR profile (SAS‐1^high^ MAS‐1^low^), giving rise to the authors proposed 15 year survival advantage. The authors showed that among individuals of similar chronological age, these IR phenotypes not only associate with immunocompetence and inflammation regulation but also directly influence healthspan and lifespan (Manoharan et al. 2025).

Salutogenesis reflects an individual's capacity to maintain function and health despite the inevitable physiological decline associated with aging. Maintaining healthspan is a delicate balance between preserving salutogenesis and countering pathogenesis. Incorporating salutogenic principles into geroscience is not only important for preventing age‐related diseases but also for preserving resilience and adaptation to stressors. This research advances our understanding of the key determinants of healthspan and lifespan, and further underscoring the importance of IR. The authors demonstrate that IR is significantly influenced by a *TCF7*‐linked trait, via a rheostat‐like mechanism, that impacts both healthspan and lifespan. Furthermore, they showed that IR degradation occurs in an age‐independent manner and can act as accelerator for aging via inflammatory stress. Salutogenic *TCF7*‐linked IR status could serve as a valuable biomarker for health, helping to identify individuals (IR‐degraders) who are most susceptible to developing the pathogenic triad. Incorporating salutogenic biomarkers could improve upon conventional biomarkers of aging, which typically focus on disease trajectories and the progression of biological aging. From an intervention standpoint, the investigation of the salutogenic biological “warranty period” reveals mid‐adulthood as the optimum time to intervene in order to optimize IR as the strong biological benefit of IR‐*TCF7* ^high^ status begins to wain by 70 years of age (Manoharan et al. 2025). This approach is vital in geroscience, where understanding the molecular and immunological basis of healthspan and longevity can guide future therapeutic interventions for treating or possibly preventing age‐related diseases. For example, identifying drugs that enhance SAS‐1 may improve immunosurveillance against pathogenic cells of aging, while interventions that limit the MAS‐1 phenotype (e.g., TNF⍺ blockers) could abate inflammaging. Overall, this work highlights the importance of salutogenesis in preventing the pathogenic triad and offers valuable insights into maintaining optimal IR to enhance longevity.

## Author Contributions

Rituparna Ghosh and Matthew J. Yousefzadeh drafted and edited the manuscript and figure.

## Conflicts of Interest

The authors declare no conflicts of interest.

## Data Availability

Data sharing is not applicable to this article as no new data were created or analyzed in this study.
